# Effects of Cultivar and Maternal Environment on Seed Quality in *Vicia sativa*

**DOI:** 10.3389/fpls.2017.01411

**Published:** 2017-08-17

**Authors:** Rong Li, Lijun Chen, Yanpei Wu, Rui Zhang, Carol C. Baskin, Jerry M. Baskin, Xiaowen Hu

**Affiliations:** ^1^State Key Laboratory of Grassland Agro-ecosystems, College of Pastoral Agriculture Science and Technology, Lanzhou University Lanzhou, China; ^2^Department of Biology, University of Kentucky, Lexington KY, United States; ^3^Department of Plant and Soil Sciences, University of Kentucky, Lexington KY, United States

**Keywords:** maternal environmental effect, seed germination, seed longevity, seed quality, seed vigor

## Abstract

Production of high quality seeds is of fundamental importance for successful crop production. However, knowledge of the effects of increased temperature resulting from global warming on seed quality of alpine species is limited. We investigated the effect of maternal environment on seed quality of three cultivars of the leguminous forage species *Vicia sativa*, giving particular attention to temperature. Plants of each cultivar were grown at 1700 and 3000 m a.s.l., and mass, germination, electrical conductivity (EC) of leakage and longevity were determined for mature seeds. Seeds of all three cultivars produced at the low elevation had a significantly lower mass and longevity but higher EC of leachate than those produced at the high elevation, suggesting that increased temperatures decreased seed quality. However, seed viability did not differ between elevations. The effects of maternal environment on seed germination strongly depended on cultivar and germination temperature. At 10 and 15°C, seeds of “Lanjian 3” produced at high elevation germinated to higher percentages and rates than those produced at low elevation, but the opposite trend was observed at 20°C. However, for seeds of “Lanjian 1” and “Lanjian 2,” no significant effect of elevation was observed in germination percentage. Our results indicate that the best environment for the production of high quality seeds (e.g., high seed mass, low EC, high seed longevity) of *V. sativa* is one in which temperatures are relatively low during seed development.

## Introduction

Successful crop production in any environment depends initially on the quality of the seeds sown. The term “seed quality” is used in agriculture to describe the overall value of a seed lot for its intended purpose (Hampton, 2002) and includes seed mass, storability, vigor, and germinability. Generally, seed quality is determined by genetic background and the environmental conditions of the mother plant during seed development (Hampton et al., 2013). It is well-known that the germination and viability of seeds may vary greatly from year to year and from one production site to another (Hampton, 2002). Much of this variation has been attributed to differences in environmental factors in space and time, including temperature (Egli et al., 2005; Shinohara et al., 2006; Pagamas and Nawata, 2008), soil moisture (Baskin and Baskin, 2014), and soil nutrients (Fenner, 1991; Baskin and Baskin, 2014).

The temperature experienced by mother plants during seed development and maturation is one of the most important environmental factors affecting seed quality components such as seed mass (Peltonen-Sainio et al., 2011), dormancy (Donohue et al., 2008; Boyko et al., 2010; Edwards et al., 2016) and germination (Gutterman, 2000). Increased temperature has been reported to decrease (Spears et al., 1997; Egli et al., 2005), not to change or increase seed mass (Peltonen-Sainio et al., 2011). In some species, plants growing at high temperatures produce seeds that are less dormant and thus have higher germination capacity than those growing at low temperature (Datta et al., 1972; Edwards et al., 2016), while in other species an inverse relation between maturation temperature and germinability has been reported (Keigley and Mullen, 1986; Ceccato et al., 2011). In view of the varied effects of temperature during seed maturation on seed quality (mentioned above), increasing temperatures resulting from future global warming (Houghton et al., 2001) is of concern to the seed production industry. However, in most studies either seed mass or germination has been used as an indicator of seed quality, which may underestimate the maternal environmental effect since environment can significantly reduce seed vigor but not germination (Hampton, 2002; Hampton et al., 2013). Further, a change in seed mass does not necessarily imply a negative effect on germination or vigor (Hampton et al., 2013). Thus, a comprehensive study of seed quality components is necessary to elucidate the role of maternal temperature on seed quality. Moreover, the seed response to maternal temperature is species (genotype and thus cultivar) specific (Shinohara et al., 2006; Baskin and Baskin, 2014). Thus, understanding the response pattern of seed quality to environmental changes at the genotype (cultivar) level is of fundamental importance for the seed industry in minimizing the risk of reductions in seed quality with global warming.

*Vicia sativa*, an annual legume species, is widely distributed throughout Eurasia and Africa. It is an economically valuable forage species that is an important source of crude protein for livestock in high elevation ecosystems due to its cold tolerance and relatively high seed production (Nie, 2011; Hu et al., 2013). On the Qing-Tibetan Plateau, *V. sativa* is mainly grown at the elevation of 2000–3500 m, and used as a fodder and seed dual-purpose crop in the local farming system. Climate change will result in higher summer temperatures and thus a longer growing season in the alpine ecosystem (Houghton et al., 2001). This increase in temperatures during the growing season could affect seed production and seed quality in *V. sativa*, thereby affecting utilization of this species. In a preliminary study, three cultivars of this species had significantly higher seed yield at 3000–3500 m a.s.l. than at 2000–2500 m a.s.l., suggesting that high temperature had a negative effect on seed production. Hu et al. (2004) showed that late (vs. early) sowing at 3000 m a.s.l. significantly decreased seed mass and increased seed leachate electrical conductivity (EC) in one of four lines of *V. sativa* in 2000 and 2001. However, in the other three lines of *V. sativa*, a significant decrease in seed quality (seed mass, germination, and leachate EC) by late sowing was observed in 2000 only. These results suggest that maternal environment may have an effect on seed quality with variation among genotypes. However, this study did not provide insight into the effects of genetic variation and environmental temperature on seed quality of *V. sativa*.

We hypothesized that increasing temperature would decrease all components of seed quality of *V. sativa* and that this change in seed quality in response to increasing temperature would differ among cultivars. To test this hypothesis, we grew three cultivars at two elevations that differed in temperature regime. Seed mass, germination, vigor, and longevity of mature seeds of the three cultivars from two production sites were determined.

## Materials and Methods

### The Cultivars and Study Area

Three cultivars of *V. sativa*, “Lanjian 1,” “Lanjian 2,” and “Lanjian 3,” were used in this study. They were bred to solve protein shortage problems for animal husbandry on the Qing-Tibetan Plateau. Seeds of these three cultivars can mature at high elevations (up to 3500 m), and generally the length of the growth period is “Lanjian 1” > “Lanjian 2” > “Lanjian 3” (**Table 1**). Seeds of the three cultivars are water permeable, i.e., do not have physical dormancy, but they do differ in degree of non-deep physiological dormancy at harvest when cultivated in the high elevation area. Thus, for example, fresh seeds of “Lanjian 3” cannot germinate at 20 and 25°C, whereas those of “Lanjian 1” and “Lanjian 2” can germinate to a high percentage at 20°C. Seeds used for sowing in this study were produced in 3000 m a.s.l.

**Table 1:** Phenology of three *Vicia sativa* cultivars grown at Yuzhong (YZ) and at Xiahe (XH).

Cultivars	Growing sites	Sowing	Emergence	First flowering	Full bloom	Seed harvest
Lanjian 1	YZ	May 14	May 20	June 25	July 5	August 13
	XH	April 30	May 27	July 10	July 16	September 2
Lanjian 2	YZ	May 14	May 21	June 25	July 4	August 8
	XH	April 30	May 26	July 5	July 12	August 25
Lanjian 3	YZ	May 14	May 22	June 24	July 2	August 2
	XH	April 30	May 24	July 1	July 5	August 18

**FIGURE 1:**
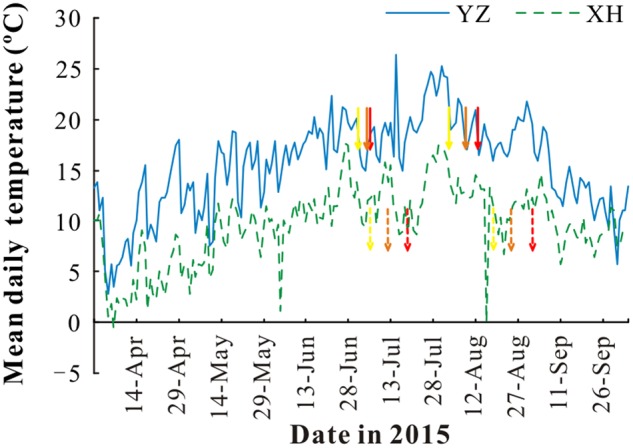
Mean daily temperature at Yuzhong (YZ) and Xiahe (XH) stations. The arrows indicate the start and end dates of seed filling of “Lanjian 1” (red), “Lanjian 2” (orange), and “Lanjian 3” (yellow) at YZ (solid line) and XH (dashed line).

To determine the effect of maternal environmental conditions especially temperature on seed quality, seeds were produced at a research station at 3000 m a.s.l. and another one at 1700 m a.s.l. Xiahe station (hereafter XH) is located at 3000 m a.s.l. (102°83′ E, 35°81′ N) on the eastern part of the Qing-Tibetan Plateau and has a mean annual temperature of 2.6°C and a mean annual rainfall of 516 mm. Yuzhong station (hereafter YZ) is located at 1700 m a.s.l. (104°10′ E, 35°57′ N) to the east of the Qing-Tibetan Plateau and has a mean annual temperature of 6.7°C and a mean annual rainfall of 350 mm (**Figure 1**). The soil in XH and YZ station are sub-alpine meadow and cultivated loessial soils, respectively, according to the Chinese Soil Classification System (Gong, 1999). The soil available nitrogen, phosphate, and potassium were 12.1 ± 0.73, 8.5 ± 0.65, 219 ± 23.7 mg/kg in XH, respectively, and 13.5 ± 0.84, 7.4 ± 0.64, 199 ± 9.0 mg/kg in YZ, respectively. Seeds of each cultivar were sown in a 0.1 ha area at the XH and YZ stations on 30 April and 14 May, 2015, respectively (**Table 1**). At each station, 10 kg of seeds were sown in rows 30 cm apart. During the growing season, irrigation was applied at both stations as needed to prevent drought stress (maintain soil moisture content above 40% field capacity), and weeds were hand-removed at monthly intervals. The dates of sowing, first seedling emergence, first flowering, full bloom, and seed harvest were recorded for each cultivar at both stations (**Table 1**). For each cultivar, several hundred ripe pods were collected at seed maturity (i.e., pods became yellow, and beginning to open) at each station. For new harvest pods, seeds were carefully removed from the fruits to avoid any mechanical damage, and four replicates of 50 seeds each were used to measure seed moisture content after oven-drying at 105°C for 72 h. Seed moisture content at harvest was ranged from 21.4 to 26.7% in XH, and from 18.8 to 25.3% in YZ. All pods were allowed to dry at room temperature (RH, 20–35%, 18–25°C) for 1 week during which seed moisture content decreased to around 10%, and then stored at 4°C until used in experiments within 2 weeks.

### Maternal Environment Effect on Seed Mass

One-thousand seed mass was determined by weighing eight replicates of 100 seeds of each cultivar from both growing sites. Seed coat mass: seed mass ratio was determined. For each seed lot, 20 seeds were individually weighed to the nearest 0.1 mg, germinated, seed coats retrieved, dried at room temperature (18–25°C, RH 30–45%) for 48 h and weighed to the nearest 0.1 mg.

### Maternal Environment Effect on Seed Germination and Viability

Fresh seeds of each cultivar from YZ and XH were incubated at 10, 15, 20, and 25°C in a 12/12 h photoperiod. For each test condition, four replicates of 50 seeds were placed in 11-cm-diameter Petri dishes on two sheets of filter paper moistened with 8 ml of distilled water. Seeds were monitored daily for germination for 14 days, and emergence of the radical to a length of at least 2 mm was the criterion for germination. Percentage of germinated, viable ungerminated, and dead (i.e., decayed) seeds was calculated at the end of the experiment. Ungerminated seeds with a firm and white embryo were assumed to be viable at the end of the experiment. No water-impermeable seeds were observed in any of the germination tests.

### Maternal Environment Effect on Seed Vigor

#### Electrical Conductivity

The EC test was performed with three replicates of 50 seeds for each seed lot (three cultivars × two sites). Each replicate was weighed and soaked in 100 mL distilled water in a 250- mL flask at 20°C for 24 h. Conductivity of the water was measured with a DST-A conductivity meter (Leici, Shanghai, China), and results were expressed in μS cm^-1^ g^-1^. At the end of the test, water-impermeable seeds (a very small portion of the seeds are water-impermeable when soaked for only 24 h) in each sample were examined, removed, surface dried, and weighed. This mass was subtracted from the initial mass of each replicate of seeds for calculation of leachate EC.

#### Accelerated Aging Test

Three replicates of 50 seeds of each seed lot were used for the accelerated aging test (AA). Each replicate was placed in a 5 cm × 5 cm wire mesh bag inside a closed 15 cm × 15 cm × 5 cm plastic box that contained 500 mL of distilled water; seeds were suspended above the water. Seeds were aged at 42°C for 24, 48, 72, 96, 120, or 144 h, after which a germination test was conducted to determine viability in light at 10°C as described above.

#### Data Analysis

Two-way analysis of variance (ANOVA) was used to test the effects of cultivar, growing site and their interaction on seed mass, coat mass, coat mass: seed mass ratio, and EC. Three-way ANOVA was used to test the effects of growing site, cultivar, germination temperature, and their interactions on seed germination rate (1/T_50_). T_50_ is defined as the time (h) to 50% germination of number of viable seeds sown, as calculated with GERMINATOR software (Joosen et al., 2010). The effects of cultivar, growing site, germination temperature and their interactions on germination were tested by fitting the data to generalized linear models (GLMs). Seed germination was a probability ranging from 0 to 1, hence we applied a binomial estimation of the model using a logit link function. Tukey’s test was used to compare means when significant differences were found. Independent *t*-tests were conducted to compare the means between growing sites at the cultivar level.

A GLM with binomial error and probit link function was fitted to the survival data with storage period as an explanatory variable. Both cultivar and growing site were included in the GLM as factors, thereby fitting the viability equation (Ellis and Roberts, 1980):

$$\begin{array}{c} v\;=\;K_{i}-(p/\sigma ) \end{array}$$

where v is the viability (in normal equivalent deviates, NED) of the seed lot after p days in storage, K_i_ initial viability (NED) of the seed lot and σ time (h) for viability to fall by 1 NED (i.e., the standard deviation of the normal distribution of seed deaths over time). All data were processed with GenStat, version 18.0 (VSN International, Ltd, Hemel Hempstead, United Kingdom).

## Results

### Maternal Environment Effect on Seed Mass

**FIGURE 2:**
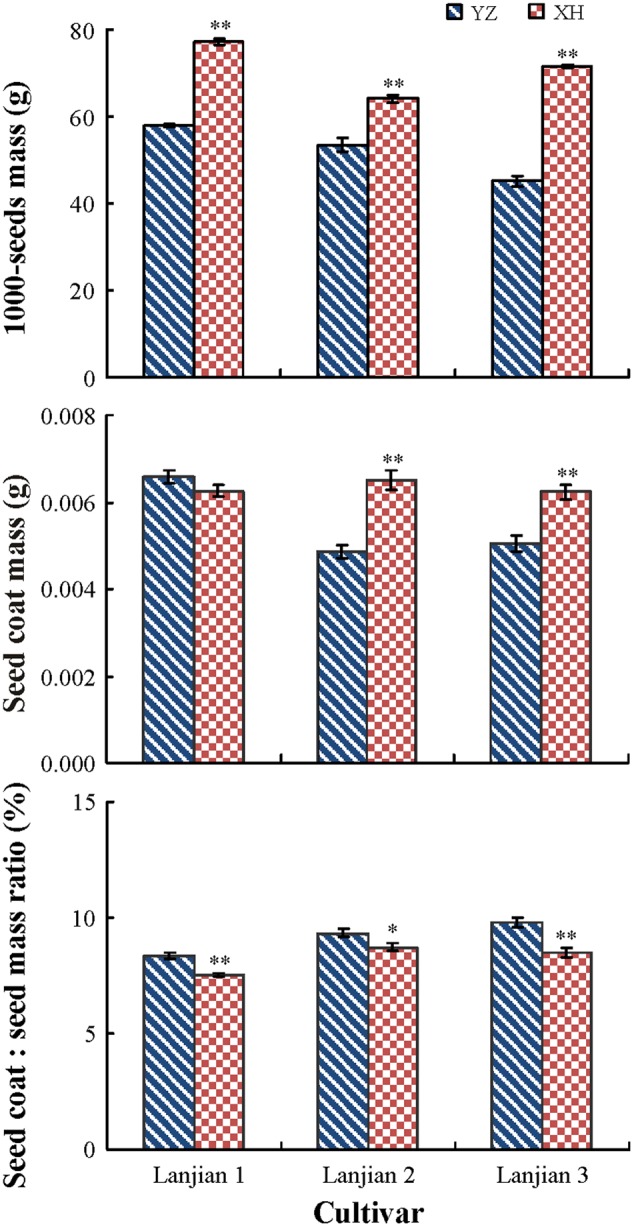
Seed mass, seed coat mass, and coat mass: seed mass ratio in three cultivars of *Vicia sativa* grown at YZ and at XH. ^∗^ and ^∗∗^ indicate significant difference between the two production sites for each cultivar at 0.05 and 0.01 levels, respectively.

Cultivar, growing site and their interaction had a significant effect on seed mass, coat mass, and coat mass: seed mass ratio (*P* < 0.001), but the interaction effect of cultivar and growing site on the coat mass: seed mass ratio was not significant (*P* = 0.09). Mass of seeds of all three cultivars from YZ was significantly lower than it was for seeds from XH, but the coat mass: seed mass ratio was higher for seeds from XH than from YZ. In contrast, seeds of “Lanjian 2” and “Lanjian 3” from YZ had a significantly lower coat mass than those from XH; however, coat mass of “Lanjian 1” did not differ between YZ and XH (**Figure 2**).

### Maternal Environment Effect on Seed Germination and Viability

**FIGURE 3:**
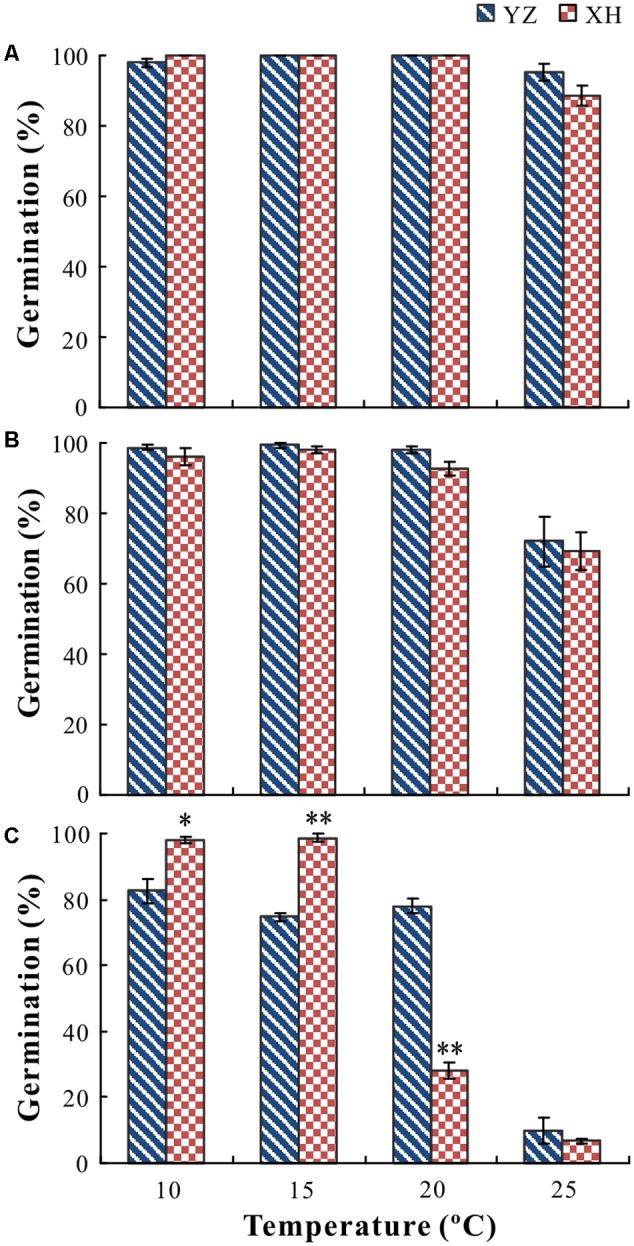
Germination percentages of seeds of *V. sativa* cultivars “Lanjian 1” **(A)**, “Lanjian 2” **(B)**, and “Lanjian 3” **(C)** produced at YZ and at XH and tested at 10, 15, 20, and 25°C. ^∗^ and ^∗∗^ indicate significant difference between two growing sites for each cultivar at 0.05 and 0.01 levels, respectively.

**FIGURE 4:**
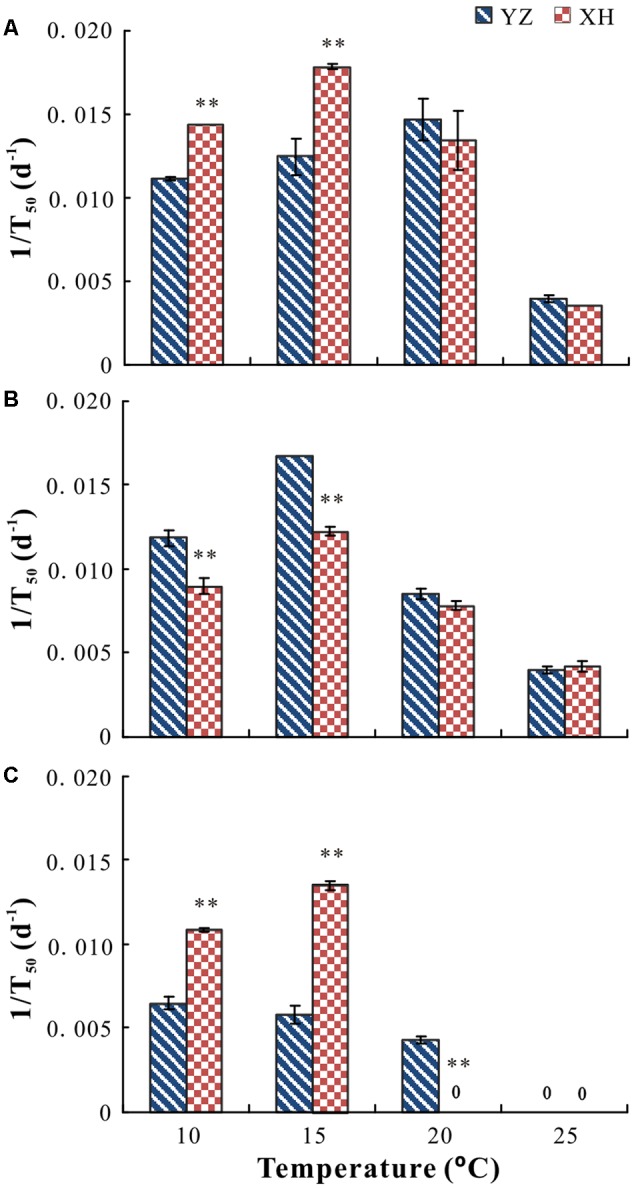
Germination rate (1/T_50_) of seeds of *V. sativa* cultivars “Lanjian 1” **(A)**, “Lanjian 2” **(B)**, and “Lanjian 3” **(C)** produced at YZ and at XH and tested at 10, 15, 20, and 25°C. ^∗^ and ^∗∗^ indicate significant difference between the two growing sites for each cultivar at 0.05 and 0.01 levels, respectively.

Cultivar, growing site, temperature and their interactions had a significant effect on germination percentage (*P* < 0.05) and rate (*P* < 0.05). For seeds of “Lanjian 1,” growing site, test temperature and their interaction had a significant effect on germination rate but not on germination percentage (**Figures 3A, 4A**). Germination rate of seeds from XH was significantly higher at 10 and 15°C than it was for seeds from YZ; however, germination rate of seeds from the two sites did not differ significantly at 20 and 25°C (**Figures 3A, 4A**).

For seeds of “Lanjian 2,” test temperature but not growing site had a significant effect on germination percentage, and seeds incubated at 25°C had a significantly lower germination percentage than those incubated at 10, 15, and 20°C (**Figure 3B**). Growing site, test temperature and their interaction had a significant effect on germination rate, and seeds from YZ exhibited a significantly higher germination rate at 10 and 15°C than those from XH. However, no significant difference in germinate rate was found for seeds from the two sites at 20 and 25°C (**Figure 4B**).

For seeds of “Lanjian 3,” growing site, test temperature and their interaction had a significant effect on germination percentage and rate (**Figures 3C, 4C**). Seeds from YZ germinated to a significantly higher percentage at 20°C than those from XH; however, the opposite trend was observed at 10 and 15°C. Consistent with this result, seeds from YZ had a significantly higher germination rate at 20°C but a significantly lower germination rate at 10 and 15°C than those from XH.

Seeds failed to germinate are viable, and no dead seed was found by the end of the experiment for all seed lots.

### Maternal Environment Effect on Seed Vigor

**FIGURE 5:**
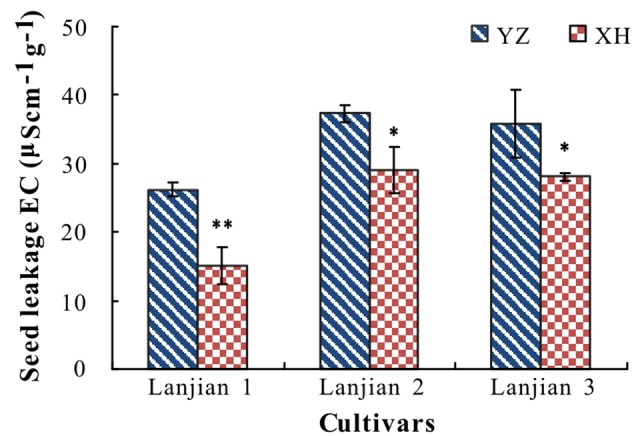
Electrical conductivity (EC, μScm^-1^ g^-1^) of seed leakage of three cultivars of *V. sativa* produced at YZ and at XH. ^∗^ and ^∗∗^ indicate significant difference between the two production sites for each cultivar at 0.05 and 0.01 levels, respectively.

Leachates from YZ seeds had a significantly higher EC than those from XH, regardless of cultivar (**Figure 5**).

**FIGURE 6:**
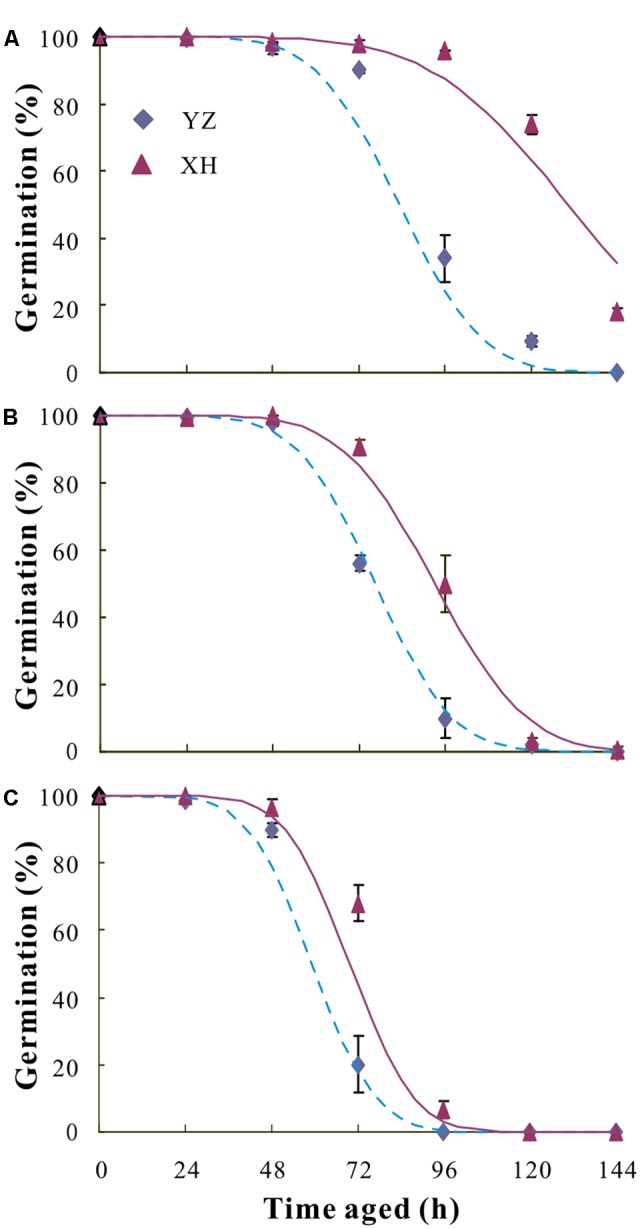
Survival curves fitted using a generalized linear model (GLM) with binomial error and probit link function for seeds of *V. sativa* cultivars “Lanjian 1” **(A)**, “Lanjian 2” **(B)**, and “Lanjian 3” **(C)** produced at YZ and at XH and aged for 0 to 144 h.

For all seed lots, seed viability declined as AA time increased (**Figure 6**). The difference in K_i_ was not significant for any of the seed lots. However, a wide variation in the estimate for σ and hence in the time taken for viability to fall to 50% was found among seed lots. Generally, seeds from XH survived much longer than those from YZ, regardless of cultivar (**Table 2**).

**Table 2:** Analysis of survival curve parameters for each seed lot determined using a generalized linear model (GLM) with binomial error and probit link function.

Cultivar	Growing site	K_i_	*SE*	σ	*SE*	*r*^2^	P_50_
Lanjian 1	YZ	4.57	0.484	18.22	1.856	0.95	83.32
	XH	4.34	0.352	30.05	3.670	0.93	130.31
Lanjian 2	YZ	4.49	0.298	17.04	0.999	0.98	76.45
	XH	4.62	0.433	20.13	2.027	0.95	93.05
Lanjian 3	YZ	4.24	0.691	13.99	1.565	0.94	59.28
	XH	4.94	0.444	14.14	1.025	0.97	69.83

*Cultivar and seed lot source (growing site) were included as factors. K_*i*_, initial viability [normal equivalent deviates (NED)] of the seed lot; σ, time (h) for viability to decrease by 1 NED; P_*50*_, time (h) for viability to decrease to 50%.*

## Discussion

In this study, seeds produced from two growing sites (1700 m vs. 3000 m a.s.l.) were used to determine the maternal environmental effect on seed quality. Since management at each site, such as fertilization, weed control and irrigation, was the same; difference in soil nutrients and moisture content were negligible. Thus, temperature was the primary environmental factor that varied between the two sites and is assumed to be the main factor accounting for differences in seed traits.

### Maternal Environment Effect on Seed Mass

Seed mass has been reported to decrease, not change or increase in response to increased temperature of the environment in which seeds were grown (Baskin and Baskin, 2014). Our study clearly showed that seeds from plants of the three cultivars grown at XH (3000 m a.s.l.) had a significantly higher seed mass than those grown at YZ, suggesting that increased temperature during seed development decreased seed mass. These results are consistent with those for *Phaseolus vulgaris* (Thomas et al., 2009), *Pisum* sp. (Lambert and Linck, 1958) and *Desmodium paniculatum* (Wulff, 1986), in that seed mass was less when plants were grown at high than at low temperature. However, seeds from plants of *Cicer arietinum* grown at 13/5°C had significantly less mass than those from plants grown at 27/18°C (Nayyar et al., 2005). Plants of *Glycine max* grown at 18/13 and 33/28°C produced seeds with significantly less mass than did those grown at 24/19, 27/22, or 30/25°C (Egli and Wardlaw, 1980).

In general, if mother plants are temperature-stressed by either high or low temperatures, seed mass is decreased (Bewley et al., 2013). A higher mass for seeds produced in XH than in YZ suggests that temperature during seed development had an effect on seed quality. Further, increased temperatures due to a decrease in elevation (or global warming) could have detrimental effects of seed quality as assessed by seed mass. High temperature during seed development may reduce seed mass due to an acceleration of seed growth rate and reduction in the duration of seed filling (Rolston et al., 1997). Indeed, for seeds produced at XH it generally took 44–52 days from full bloom to seed maturity, whereas those produced at YZ took only 31–39 days (**Table 1**). Firincioğlu et al. (2010) reported that seeds from plants of *V. sativa* grown from seeds sown in autumn had a longer seed filling period and higher seed mass than those from plants grown from seeds sown in spring. On the other hand, plants grown at YZ produced more pods (and seeds) than those grown in XH (data not shown). Thus, an alternative explanation for the higher dry mass of seeds from XH than those from YZ is that seeds from the former site had less competition for photosynthates than those from the latter site.

The maternal environment can affect total seed mass by altering mass of the seed coat, embryo, endosperm, or some combination thereof (Lacey et al., 1997). Seeds from YZ had a significantly higher embryo mass (seed mass minus seed coat mass) and seed coat mass than those from XH, suggesting that high temperature affects seed mass through both the embryo and seed coat. However, the seed coat: seed mass ratio was increased by high temperature, implying that high temperature may favor seed coat development. Similarly, seeds of soybean (Keigley and Mullen, 1986) and *Chenopodium quinoa* (Ceccato et al., 2015) developed a thicker coat under high than under low temperature. However, Lacey et al. (1997) found that high temperature decreased the seed coat: seed mass ratio of *Plantago lanceolata* but not the embryo mass or endosperm mass.

### Maternal Environment Effect on Seed Germination

For several plant species, it has been reported that seeds produced under warm conditions have a higher germination percentage than those produced under cool conditions (Baskin and Baskin, 2014). Likewise, seeds of “Lanjian 3” produced at YZ germinated to a significantly higher percentage at 20°C, but not at 10 or 15°C, than those produced at XH. However, for the other two cultivars in our study maternal environment had no effect on germination percentage, suggesting that the effect of maternal environment on seed germination was strongly dependent on cultivar. Ceccato et al. (2015) also found that late sowing increased embryo dormancy in accession Chadmo of *Chenopodium quinoa* but not in accession 2-Want. The germination responses of some genotypes in *Arabidopsis thaliana* responded weakly to maternal temperature, but for others the response was highly plastic (Burghardt et al., 2016). Thus, genotype differences in capacity to respond to increasing temperature suggests that there is a possibility of developing new cultivars of crop species that are adapted to higher global temperatures.

The maternal effect on germination percentage and rate in our study was largely due to the germination test temperature. For example, seeds of “Lanjian 3” produced at XH had a higher germination percentage and rate at low temperature than those produced from YZ. However, seeds of “Lanjian 3” produced at YZ had a higher germination percentage and rate at high temperature than those produced at XH. Plett and Larter (1986) found that the maturation temperature resulting in the highest germination of *X Triticosecale* and *Triticum aestivum* depended on the temperature at which the seeds were tested. Further, seeds produced by plants of *Colobanthus quitensis* grown at 5/2°C germinated to a higher percentage at 10 and 15°C but to a lower percentage at 20 and 25°C than those produced by plants grown at 11/15°C (Sanhueza et al., 2016). These contradictory results from previous research may be attributed partly to the seed testing conditions. Thus, testing germination over a range of environmental conditions is necessary to determine the effect of maturation temperature (Gutterman, 1992).

The measurement/determination of seed quality is less consistent when seeds are tested at various conditions, suggesting that seed germination parameters may not be a good indicator as seed quality, except when germination is used as a method for determining seed viability. The combined results on seed mass, EC and seed longevity (AA) obtained in our study show that increasing the temperature during seed production decreased seed quality in all three cultivars. However, this conclusion could not be derived only from germination tests, and in some cases the results of germination tests lead to a wrong conclusion about seed quality.

### Maternal Environment Effect on Seed Vigor and Longevity

High temperatures during seed production can increase seed cell membrane damage (Nilsen and Orcutt, 1996; Shinohara et al., 2006) and thus increase electrolyte leakage from seeds. Consistent with this, seeds from plants of the three cultivars grown in YZ exhibited a significantly higher EC than those from plants grown in XH.

The use of the AA test has been proposed for evaluation of both seed vigor and seed storability (Copeland and McDonald, 1995; Samarah, 2006). Environmental conditions during seed development and maturation are known to affect the longevity of seeds. For example, climate warming has increased seed longevity in four alpine species as measured by an AA test (Bernareggi et al., 2015). However, according to results from AA tests, mean seed longevity of *Wahlenbergia tumidifructa* seeds produced under warm-wet conditions was significantly lower than that of seeds produced under cool wet-conditions (Kochanek et al., 2010). Consistent with this, the results of our AA test show that the mean seed longevity of the three cultivars produced at YZ (higher temperature) was lower than it was for those produced at XH (lower temperature).

Differences in longevity of seeds produced under various environmental conditions are due to differences in initial viability (K_i_) and the distribution of individual seed deaths over time (σ) (Kochanek et al., 2009). Our study also revealed that the differences in *P_50_* were attributable to the parental growth environment causing changes in σ but not in K_i_. This is consistent with the results for *W. tumidifructa* in which σ was primarily influenced by the temperature experienced by mother plants and K_i_ mainly by soil moisture (Kochanek et al., 2009, 2010).

It is also worth noting that the reduction in seed longevity by increased environmental temperature was closely related to the cultivar. For example, the increased growth temperature significantly reduced σ in “Lanjian 1” and “Lanjian 2” but not “Lanjian 3.” Moreover, “Lanjian 1” had a much higher mean seed longevity (AA) than “Lanjian 2” and “Lanjian 3” at each growing site. The variation among cultivars in seed longevity further suggests that it will be possible to select new cultivars that will be suitable for cultivation at various elevations as global warming increases.

In summary, we conclude that the predicted increase in environmental temperature will lead to lower seed quality, particularly seed vigor, and storage capability. Germination percentages also will change. However, if not caused by reduction in seed viability, the change does not necessarily imply a negative effect on seed vigor, which is strongly dependent on germination test temperature. Moreover, the variation in seed quality among cultivars in response to increasing growth temperature detected in our study suggests that selection of new cultivars is a key way to cope with loss of seed quality in response to increased global warming.

## Author Contributions

XH and YW conceived the topic. RL and LC performed the experiments. RL and RZ analyzed all statistical data. RL and XH wrote the manuscript. JB and CB revised the manuscript.

## Conflict of Interest Statement

The authors declare that the research was conducted in the absence of any commercial or financial relationships that could be construed as a potential conflict of interest.
